# Intra-articular injections for shoulder arthritis in adults: a systematic review

**DOI:** 10.1186/s40001-025-03423-4

**Published:** 2025-11-07

**Authors:** Filippo Migliorini, Luise Schäfer, Virginia Masoni, Fabrizio Rivera, Gennaro Pipino, Nicola Maffulli

**Affiliations:** 1 Department of Trauma and Reconstructive Surgery, University Hospital of Halle, Martin-Luther University Halle-Wittenberg, Ernst-Grube-Street 40, 06097 Halle (Saale), Germany; 2 Department of Orthopaedic and Trauma Surgery, Academic Hospital of Bolzano (SABES-ASDAA), Via Lorenz Böhler 5, 39100 Bolzano, Italy; 3 Department of Life Sciences, Health, and Health Professions, Link Campus University, Via del Casale Di San Pio V, 00165 Rome, Italy; 4 Department of Orthopaedic and Trauma Surgery, Eifelklinik St. Brigida, Kammerbruchstr. 8, 52152 Simmerath, Germany; 5 Department of Orthopaedics and Traumatology, University of Turin, Via Zuretti, 29, 10126 Turin, Italy; 6 Department of Orthopaedics and Traumatology, Ospedale SS Annunziata, ASL CN1, Via Ospedali, 9, 12038 Savigliano, Italy; 7 Department of Orthopaedics, Villa Erbosa Hospital, IRCCS Ospedale S. Raffaele, Milan, Italy; 8 Department of Trauma and Orthopaedic Surgery, Faculty of Medicine and Psychology, University La Sapienza, 00185 Rome, Italy; 9 School of Pharmacy and Bioengineering, Keele University, Faculty of Medicine, Stoke on Trent, ST4 7QB UK; 10 Centre for Sports and Exercise Medicine, Barts and the London School of Medicine and Dentistry, Mile End Hospital, Queen Mary University of London, London, E1 4DG UK

**Keywords:** Shoulder arthritis, Intra-articular therapy, Hyaluronic acid, Corticosteroids, Platelet-rich plasma, Bone marrow aspirate concentrate, Mesenchymal stem cells

## Abstract

**Background:**

The management of glenohumeral osteoarthritis (GHOA) is challenging, particularly in patients who are not eligible for surgery. In recent years, several injectable therapies, including hyaluronic acid (HA), corticosteroids (CCs), platelet-rich plasma (PRP), bone marrow aspirate concentrate (BMAC), and mesenchymal stem cells (MSCs), have emerged as potential options for managing pain and improving joint function. This systematic review aims to summarise the current evidence on infiltrative strategies to manage GHOA in adults.

**Methods:**

This review followed the PRISMA 2020 guidelines. PubMed, Web of Science, and Embase were systematically searched in May 2025. All clinical studies investigating infiltrative strategies to manage shoulder arthritis in adults were considered for inclusion. Only studies with a minimum follow-up of six months were included. The methodological quality of the included studies was assessed using the Cochrane RoB2 tool for randomised controlled trials (RCTs) and the ROBINS-I tool for non-randomised studies.

**Results:**

Data from 1125 patients (1126 shoulders) were analysed. The mean age of the patients was 63.4 ± 5.8 years, and 34.1% (384 of 1125 patients) were women. The most commonly studied intra-articular treatments included HA and CCs. The rate of surgery for persistent symptoms or functional impairment was 3.2% (35 of 1079 reported procedures). The overall rate of complications was 7.2% (56 of 780 reported procedures).

**Conclusion:**

Infiltrative management can provide symptomatic relief in adults with GHOA. Current evidence supports the potential role of different injectable therapies, with hyaluronic acid demonstrating consistent, though modest, benefits. In contrast, the evidence for orthobiologics remains limited, mainly because of heterogeneity in study design, outcome measures, and patient characteristics. High-quality comparative trials with long-term follow-up are required to establish optimal treatment strategies and to identify patient subgroups most likely to benefit from specific interventions.

**Supplementary Information:**

The online version contains supplementary material available at 10.1186/s40001-025-03423-4.

## Introduction

Glenohumeral osteoarthritis (GHOA) is a common cause of shoulder pain and functional impairment [1, 2], affecting an estimated 5% to 17% of patients presenting with shoulder-related concerns [3–5]. Age and obesity, well-established risk factors for hip and knee osteoarthritis (OA), also contribute to GHOA. Still, their impact appears less pronounced, given the shoulder joint's distinct biomechanical and anatomical features, rendering its pathogenesis more complex and multifactorial [6, 7]. Large-scale analyses indicate that age-related primary GHOA is approximately 10 times more common than secondary causes in the general population [8, 9]. However, this trend reverses in patients under 50, in whom trauma, glenohumeral instability, or occupational overuse (e.g. in overhead athletes or heavy manual labourers) are more often implicated [10, 11]. Additional structural pathologies, such as rotator cuff arthropathy, may also accelerate degenerative changes [5, 12]. Diagnosing GHOA can be challenging given the overlapping symptoms with other shoulder pathologies, including adhesive capsulitis [13, 14]. In the absence of a universally accepted clinical definition, radiographic grading according to the Samilson-Prieto classification, initially designed for post-dislocation arthritis, remains the most widely used tool for assessing GHOA severity [15]. First-line treatment usually involves nonoperative strategies, especially in younger or less symptomatic patients, including lifestyle modifications, nonsteroidal anti-inflammatory drugs (NSAIDs), physiotherapy, and intra-articular injections [16, 17]. These approaches aim to reduce pain, improve function, and delay or prevent the need for surgical intervention [17–20]. However, the optimal management of GHOA, particularly in patients under 50, remains a subject of ongoing debate [21]. Among nonoperative treatments, infiltrative strategies have gained particular interest given their potential disease-modifying effects and the increasing availability of biological agents [22, 23]. In this regard, corticosteroids (CCs) and hyaluronic acid (HA) are the most commonly employed agents. However, newer biological agents, such as platelet-rich plasma (PRP), bone marrow aspirate concentrate (BMAC), and mesenchymal stem cells (MSCs), have recently gained attention [24–27]. These compounds differ substantially in their mechanisms of action and therapeutic objectives [28–31]. Whilst CCs offer short-term symptom relief through anti-inflammatory effects, HA aims to restore joint lubrication and provide chondroprotection [32, 33]. Despite their widespread use [34–36], current evidence for these treatments in GHOA is inconclusive, especially for long-term outcomes [37]. Data on PRP in GHOA are limited but promising, with more robust evidence available in related conditions, such as rotator cuff tears [38, 39]. Similarly, the use of cell-based biologics, such as BMAC and MSCs, remains experimental, with no conclusive clinical efficacy established to date [40, 41].

Given the heterogeneity and evolving landscape of infiltrative options for GHOA, this systematic review aims to critically assess and synthesise the current clinical evidence on infiltrative strategies for GHOA in adults.

## Methods

### Eligibility criteria

All clinical studies investigating infiltrative management for GHOA in adults were considered. Eligible articles were written in English, German, Italian, French, or Spanish. Studies with a minimum follow-up of six months were included. According to the Oxford Centre for Evidence-Based Medicine [42], studies with levels of evidence I to III were eligible. Therefore, randomised controlled trials (RCTs), prospective and retrospective cohort studies, case–control studies, and cross-sectional studies were included in the analysis. Exclusion criteria comprised reviews, case reports, letters, expert opinions, editorials, animal studies, in vitro studies, and biomechanical or cadaveric research.

### Search strategy

This systematic review was developed in accordance with the PRISMA 2020 guidelines [43]. The search strategy targeted studies according to the following criteria:

- Problem: glenohumeral arthritis in adults;
- Intervention: infiltrative management;
- Timing: minimum six months of follow-up;

In May 2025, the following databases were accessed: PubMed, Web of Science, and Embase. No filters or time constraints were set. The Medical Subject Headings (MeSH) used for the database search are listed in the Appendix.

### Selection and data collection

Two independent reviewers (F.M. and L.S.) carried out the screening process. All titles identified through the database search were manually reviewed. When a title appeared relevant, the corresponding abstract was assessed. Full-text articles were retrieved for studies that met the inclusion criteria based on title and abstract screening. If the full text was unavailable, the study was excluded from analysis. Additionally, the reference lists of the included full-text articles were checked to identify further eligible publications. Any discrepancies between the reviewers were resolved through discussion, and, if necessary, a third senior author (V.M.) provided the final judgement.

### Data items

Two authors (F.M. and L.S.) extracted data from the included studies. The following information was systematically collected: first author, year of publication, journal, study design, level of evidence, duration of follow-up, number of patients and treated shoulders, and mean patient age. Details on the type of intervention, outcome parameters, and any reported complications or reoperations were also documented. All data were organised using Microsoft Excel (version 16.0, Microsoft Corporation, Redmond, WA, USA).

### Assessment of the risk of bias

The risk of bias was assessed in accordance with the recommendations outlined in the Cochrane Handbook for Systematic Reviews of Interventions [44]. Two reviewers (F.M. & L.S.) independently evaluated the risk of bias in the included studies. Disagreements were solved in consultation with a third senior author (V.M.). RCTs were assessed using the revised Risk of Bias assessment tool (RoB2) [45, 46] of the Cochrane tool for assessing Risk of Bias in randomised trials (RoB) [47]. The following endpoints were evaluated: bias arising from the randomisation process, bias based on deviations from intended interventions, bias due to missing outcome data, bias in the measurement of the outcome, and bias in the selection of the reported result.

Non-randomised controlled trials (non-RCTs) were evaluated using the Risk of Bias in Non-Randomised Studies of Interventions (ROBINS-I) tool [48]. Seven domains of potential bias in non-RCTs were assessed. Two domains assessed the possible confounding variables and the nature of patient selection before the start of the comparative intervention. Bias in the classification during the intervention was evaluated by a further domain. The final four domains were used to assess the methodological quality after the intervention comparison had been implemented, taking into account deviations from previously intended interventions, missing data, erroneous measurement of outcomes, and bias in the selection of reported outcomes. The figure of the ROBINS-I was elaborated using the Robvis Software (Risk-of-bias VISualization, randomisation Riskofbias.info, Bristol, UK) [49].

### Synthesis methods

The statistical analysis was performed by the main author (F.M.) using IBM SPSS Statistics (version 25.0; IBM Corp., Armonk, NY, USA). The approach was based on the general recommendations of the Cochrane Handbook for Systematic Reviews of Interventions [50]. Descriptive statistics were used to summarise the extracted data. Continuous variables were reported as arithmetic means and standard deviations. Dichotomous variables were presented as absolute frequencies (events/observations).

## Results

### Study selection

**Fig. 1:**
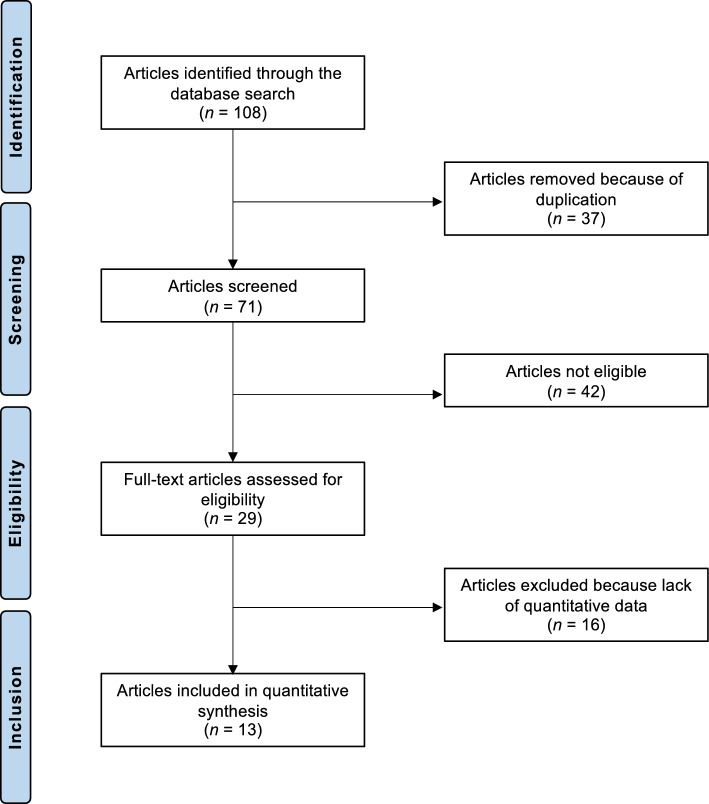
PRISMA flow chart of the literature search

The literature search initially identified 108 potentially relevant studies. After removing 37 duplicates, 71 articles underwent the first title and abstract screening, and then they were reviewed in detail. Of these 42 articles were excluded for the following reasons: inappropriate study type and design (*N* = 18), methodological limitations (*N* = 4), inadequate or unclear follow-up (*N* = 3), overlapping patient populations (*N* = 2), indication not related to degenerative hyaline cartilage lesions of the glenohumeral joint (*N* = 5), focus on open surgical or arthroplasty procedures such as total or reverse shoulder replacement (*N* = 8), or the language of publication was outside the range of the authors’ proficiency (*N* = 2). An additional 16 studies were excluded after a full-text examination due to the lack of quantitative outcome data. Ultimately, 13 clinical studies were included in the final quantitative synthesis. These comprised five RCTs, six prospective cohort studies, and two retrospective studies. The selection process is reported in Fig. 1.

### Risk of bias assessment

**Fig. 2:**
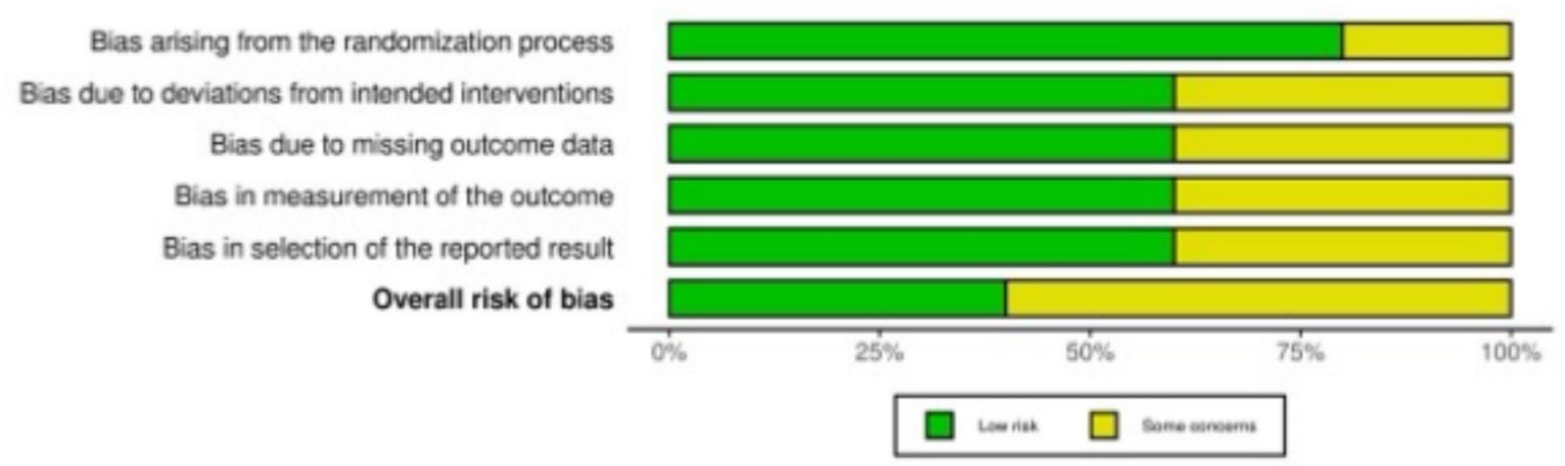
The RoB2 of the included RCTs

Five of the 13 studies (38.5%) included in this systematic review were RCTs, and were assessed using the Cochrane Risk of Bias 2 (RoB2) tool. The randomisation process was clearly described and adequately implemented in all five RCTs, resulting in a low risk of bias in this domain. Most trials followed well-defined treatment protocols; however, two studies raised some concerns regarding deviations from intended interventions. Concerns related to outcome measurement were identified in two trials, primarily from a lack of blinded outcome assessment. One study raised concerns from missing data, whilst selective reporting was considered a low risk in most studies, with only two trials showing some concerns. Overall, three of the five RCTs were judged to have some concerns regarding risk of bias, whilst two were rated as having a low risk. These findings reflect an acceptable level of methodological quality amongst the RCTs, despite minor limitations in individual domains (Fig. 2).

**Fig. 3:**
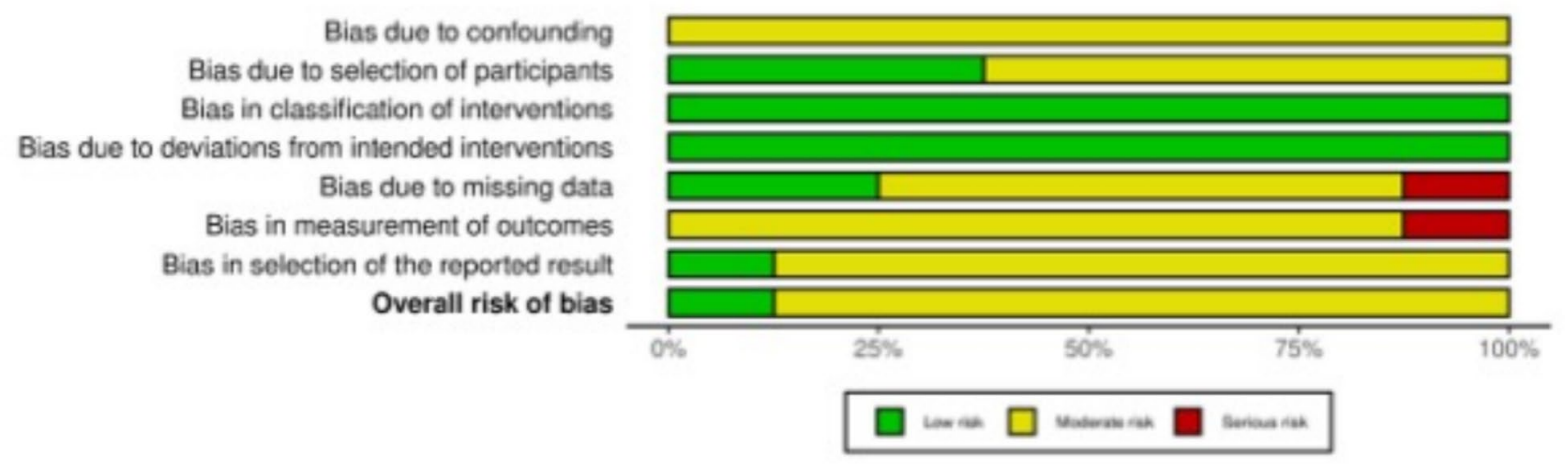
The ROBINS-I

Of the 13 studies included in this review, 61.5% (8 of 13) were non-randomised and were therefore assessed using the ROBINS-I tool. In the domain of confounding, all eight studies were judged to have a moderate risk of bias, reflecting a common limitation of non-randomised designs, namely, the limited ability to control for potential confounding factors. In contrast, the risk of bias in participant selection was rated as moderate or low in all studies, indicating that recruitment and eligibility criteria were generally appropriate. All studies were rated as having low risk of bias in both the classification of interventions and adherence to the intended intervention protocols, suggesting that treatments were clearly defined and consistently delivered. Post-intervention domains revealed further concerns: several studies had moderate to serious risks associated with missing outcome data or limitations in outcome measurement, often related to retrospective designs, subjective assessments, or incomplete follow-up. The domain addressing selection of reported results was typically rated as moderate risk, mainly from the absence of prospective trial registration or publicly available protocols. Overall, seven of the eight non-randomised studies were assessed as having a moderate overall risk of bias, whilst one study was rated as low risk. These results indicate an acceptable level of methodological quality across the included non-RCTs, albeit with typical limitations in confounding and post-intervention assessment (Fig. 3).

### Study characteristics and results of individual studies

The 13 studies included data from 1125 patients (1126 treated shoulders). The average age of the patients was 63.4 ± 5.8 years. A total of 34.1% (384 of 1125 patients) were women. A comprehensive overview of study characteristics and patient demographics is presented in Table 1.

**Table 1:** Generalities and patient demographics of the included studies

Author and year	Journal	Design	Follow up (months)	Treatment and number of injections	Patients (*n*)	Shoulders (*n*)	Mean age	Women (*n*)
Blaine et al. 2008 [51]	*J Bone Joint Surg Am*	RCT	6	HA (*n* = 5) and PBS (*n* = 3)	136	136	NR	NR
				HA (*n* = 5) and PBS (*n* = 5)	129	129		
				PBS (*n* = 5)	133	133		
Brander et al. 2010 [52]	*PM R*	Prospective	6	HA (*n* = 5)	36	36	67.0	20
Centeno et al. 2015 [53]	*J Pain Res*	Prospective	12	BMC (*n* = 1)	34	34	52.1	7
Dwyer et al. 2021 [41]	*Arthrosc Sports Med Rehabil*	RCT	12	BMA (*n* = 1)	25	13	61.6	5
				CCs (*n* = 1)		12	53.8	3
Fan et al. 2022 [54]	*Regen Med*	Prospective	12	MFAT (*n* = 1)	13	13	64.2	9
Kirschner et al. 2022 [55]	*Clin J Sport Med*	RCT	12	HA (*n* = 1)	36	36	64.4	18
				LP-PRP (*n* = 1)	34	34	69.1	20
Kwon et al. 2013 [56]	*J Shoulder Elbow Surg*	RCT	6	HA (*n* = 1)	133	133	65.9	53
				PBS (*n* = 1)	130	130	65.7	63
Merolla et al. 2011 [57]	*Musculoskelet Surg*	Retrospective	6	HA (*n* = 3)	51	51	61.0	38
				CCs (*n* = 3)	33	33	63.0	23
Metzger et al. 2011 [58]	*J Shoulder Elbow Surg*	Prospective	12	CCs (*n* = 3)	29	30	66.1	14
Monti et al. 2025 [59]	*Reumatismo*	Retrospective	7	HA (*n* = 3)	40	40	67.2	19
Noël et al. 2009 [60]	*Joint Bone Spine*	Prospective	6	HA (*n* = 1)	17	17	58.3	8
				HA (*n* = 2)	16	16	55.0	7
Silverstein et al. 2007 [61]	*Am J Sports Med*	Prospective	6	HA (*n* = 3)	30	30	62.0	10
Tortato et al. 2022 [62]	*Acta Ortop Bras*	RCT	6	HA (*n* = 1)	38	38	72.7	36
				CCs (*n* = 1)	32	32	72.2	31

BMA: bone marrow aspirate; CCs: corticosteroids; HA: hyaluronic acid; PRP: platelet-rich plasma; LP-PRP: leukocyte-poor platelet-rich plasma; PBS: phosphate-buffered saline; RCT: randomised controlled trial; TSA: total shoulder arthroplasty; NR: not reported

### Complications

The rate of surgery for persistent symptoms or functional impairment was 3.2% (35 of 1079 reported procedures). The overall rate of complications was 7.2% (56 of 780 reported procedures). An overview of revision procedures and complications is provided in Table 2.

**Table 2:** Reported revisions and complications of the included studies

Author and year	Treatment and number of injections	Surgery (*n*)	Complications (*n*)	Remarks on complications and revision surgeries
Blaine et al. 2008 [51]	HA (*n* = 5) and PBS (*n* = 3)	0	0	No surgical revisions required. No product-related serious adverse events. Most common side effects included injection-site pain and arthralgia, all mild and transient
	HA (*n* = 5) and PBS (*n* = 5)			
	PBS (*n* = 5)			
Brander et al. 2010 [52]	HA (*n* = 5)	0	3	3 patients had transient post-injection shoulder pain; no inflammatory or serious adverse events
Centeno et al. 2015 [53]	BMC (*n* = 1)	NR	5	3 cases of pain (possibly related), 1 cardiac event, 1 other (both unlikely related); no serious adverse events reported
Dwyer et al. 2021 [41]	BMA (*n* = 1)	0	0	No complications or revision surgeries were observed in either group during the 12-month follow-up
	CCs (*n* = 1)	0	0	
Fan et al. 2022 [54]	MFAT (*n* = 1)	NR	NR	No adverse events or revision surgeries were explicitly reported for shoulders
Kirschner et al. 2022 [55]	HA (*n* = 1)	16	1	Sixteen patients underwent total shoulder replacements following their study injections. Ten had surgery during the study period, and six had surgery after their 12-month follow-up
	LP-PRP (*n* = 1)		1	
Kwon et al. 2013 [56]	HA (*n* = 1)	0	NR	Minor adverse events, primarily arthralgia and musculoskeletal pain, were reported in both groups. No serious treatment-related complications or revision surgeries occurred during the 26-week trial period
	PBS (*n* = 1)	0	NR	
Merolla et al. 2011 [57]	HA (*n* = 3)	5	7	In the Hylan group, five underwent surgery (4 TSA, 1 arthroscopy); two minor events occurred in the steroid group
	CCs (*n* = 3)	0	2	
Metzger et al. 2011 [58]	CCs (*n* = 3)	12	NR	No adverse events reported; 12 shoulders required repeat injection or arthroplasty within 12 months
Monti et al., 2025 [59]	HA (*n* = 3)	1	NR	No adverse events were reported; one patient was referred for surgery for persistent symptoms
Noël et al., 2009 [60]	HA (*n* = 1)	0	10	No serious treatment-related adverse events or revision surgeries were reported
	HA (*n* = 2)			
Silverstein et al., 2007 [61]	HA (*n* = 3)	1	21	21 adverse events, none device-related; 1 patient underwent shoulder arthroplasty for persistent pain
Tortato et al. 2022 [62]	HA (*n* = 1)	0	4	Adverse events were limited to transient injection-site pain, and no revisions were required
	CCs (*n* = 1)	0	2	

HA: hyaluronic acid; TSA: total shoulder arthroplasty; AE: adverse events; NR: not reported

## Discussion

Based on the main findings of the present systematic review, infiltrative strategies may provide symptomatic relief in adults with GHOA. Current evidence supports the potential benefits of various injectable therapies, including HA, CCs, PRP, BMAC, and microfragmented adipose tissue, for the management of GHOA. Although many studies report improvements in pain and functional outcomes, the results remain heterogeneous given differences in study design, outcome measures, and patient characteristics. HA appears to offer consistent, yet modest, benefits, whereas biological therapies represent promising alternatives, particularly for younger or more active patients. Nevertheless, high-quality comparative studies with long-term follow-up are essential for defining optimal treatment strategies and identifying patient subgroups most likely to benefit from specific interventions.

Despite the lack of a universal consensus regarding the management options, the current literature tends to agree on a first-line conservative therapy consisting of lifestyle modification, NSAIDs, physical therapy, and infiltrative options [16, 17, 24, 63]. A combination of the above-mentioned is usually supported to maximise its efficacy [17, 24]. Surgery is needed in case of failure of conservative management or when severe symptoms are present [16, 17]. Lifestyle modification, sport, and workplace adaptations should be implemented, especially for disciplines involving heavy weightlifting and overhead activities [24]. Moreover, physical therapy should be initiated as soon as possible [17], as non-pharmacological options are more efficacious when started before the development of joint contracture or atrophy [24].

Intra-articular injections should be seen as a conservative management strategy [16, 17, 24]. Many typologies exist, ranging from the most common CCs and HA to platelet-rich compounds and the latest biologics, such as BMAC and MSCs [16, 17, 24]. Acting through different mechanisms, each compound has specific advantages and limitations, with no clear superiority of one compound over the others [16, 17, 24, 63, 64]. When performing intra-articular injections, several approaches, including the anterior, posterior, and supraclavicular approaches, can be employed [65]. When evaluating accuracy, no statistically significant difference was observed amongst the three. Tortato et al. [16, 17, 24] employed a posterior approach as it is the arthroscopic portal routinely used in surgical practice.

CCs are primarily employed intra-articularly for their local anti-inflammatory properties [16, 17, 24]. Different formulations exist; water-soluble compounds, such as dexamethasone, rapidly disperse from the joint and are best used in extra-articular conditions [16]. On the other hand, lower water solubility allows higher synovial concentrations and fewer systemic side effects [16, 17, 24]. However, CCs do not influence the progression of joint degeneration, and it can actually negatively impact the cartilage and accelerate GHOA [17, 24, 66]. For this reason, the current literature does not recommend more than three injections [17, 24]. Of the included studies in this systematic review, Metzger et al. [16, 17, 24] described the efficacy of a single, image-guided CC injection for GHOA with improvements in shoulder function and pain up to 4 months after injection. Data from a previous review by Gross et al. [16] reported only minor side effects, including transient pain at the injection site, facial flushing, and skin and subcutaneous fat atrophy, with the most serious complication being septic arthritis [16]. Systemic effects, such as hypothalamic–pituitary–adrenal axis suppression and hyperglycemia, are described, and greater attention should be paid to diabetic patients where raised intraocular pressures could occur [16, 17, 24]. For these reasons, the American Academy of Orthopaedic Surgeons (AAOS) reports that the use of injectable CCs for GHOA is inconclusive [16, 24, 67]. Tortato et al. [16, 17, 24] reported increased satisfaction and pain reduction after both HA and CCs injections in patients with GHOA in the absence of complete rotator cuff injury, with, however, better and longer-lasting effects in the HA group. Dwyer et al. [16, 17, 24] observed greater improvements in QuickDASH, EQ-5D-5L pain and health scores with a single intra-articular BMCA compared to patients treated with CCs at 12 months. However, CCs have been demonstrated to be beneficial for other shoulder pathologies, with a recent meta-analysis reporting better outcomes with their early use in patients with frozen shoulders lasting less than one year [68, 69].

The second most commonly injected compound is intra-articular HA. It acts both mechanically and biologically [16, 17, 24]. Given its viscoelastic properties, it increases the viscosity of the synovial fluid, thereby improving lubrication and reducing the friction coefficient [16, 17, 24, 70]. At the same time, it also offers a chondroprotective effect [16, 17, 24]. A recent targeted review of the literature reports its effects on chondroprotection and nociception in knee OA [70]. HA improves anabolic biomarkers such as collagen types II, IX, and XI, and leads to a decrease in catabolic biomarkers, including matrix metalloproteinases [70]. Moreover, it lowers soluble inflammatory mediators, such as interleukin 1β and 6, and tumour necrosis factor α, and exogenous administration of HA may facilitate the synthesis of intrinsic proteoglycans and glycosaminoglycans [70]. Several formulations exist, and HA has been mainly described as having clinical benefits in rotator cuff tendinopathy [71]. Noël et al. [71] described HA injections as a safe, feasible, and probably effective treatment in patients with shoulder osteoarthritis and an intact rotator cuff. In this regard, even Brander et al. [71] reported improved pain and function after two injections of HA, regardless of the presence or absence of rotator cuff pathology, for up to 6 months after treatment. For instance, Zhang et al. [72], who investigated the effect of intra-articular HA injection for GHOA, underlined its safety and pain improvement, with pain amelioration also observed in the control group, indicating a notable placebo effect. Nonetheless, the current evidence is controversial concerning its use in GHOA, with the AAOS supporting no benefit from HA in treating this pathology, indicating just added costs without additional benefits [24, 67]. Lastly, HA is not without side effects, such as transient joint pain [16, 72], which, however, usually subsides without sequelae as reported by Brander et al. [71] in whose study three participants experienced pain for a few days after injection.

The other groups of intra-articular injections include biologics such as PRP, BMAC, and MSCs [73]. PRP products have been increasingly used in shoulder pathologies [24, 73, 74]. The increase in their use derives from the positive effects demonstrated in knee OA [24, 75, 76]. Indeed, Bensa et al. [75] indicated that PRP offered clinically relevant functional progress and pain improvement compared with a placebo for treating knee OA. PRP has anabolic, anti-inflammatory, and immunomodulatory properties [24, 73, 75]. It has been extensively studied in treating rotator cuff damage, as PRP can be added to sutures to enhance the healing process [25]. Tang et al. [25] evaluated the use of PRP during arthroscopic surgeries for rotator cuff injuries and compared the success of leukocyte-poor PRP with that of leukocyte-rich PRP. In this context, PRP can be categorised into leukocyte-poor PRP and leukocyte-rich PRP based on the amount of white blood cells (WBCs), where leukocyte-poor PRP is more suitable for situations requiring a reduced inflammatory response, and leukocyte-rich PRP results in an augmented inflammatory response [25]. In this systematic review, Kirschner et al. [25] compared the efficacy of leukocyte-poor platelet-rich plasma (LP-PRP) injection versus HA for GHOA in a double-blind randomised controlled trial. Despite significant improvements in pain and function after both treatments, no differences were found between the two groups [25]. Indeed, as far as GHOA is concerned, the literature agrees that there is no evidence to support the use of PRP [17, 24, 63, 73]. Recently, BMAC and MSCs have garnered attention [17, 24, 73, 77]. BMAC has been introduced for knee cartilage defects and OA, as reported by Mavrogenis et al. [77], since it stimulates angiogenesis and possesses anti-inflammatory properties [17, 24, 77]. Either the iliac crest, the tibia, or the calcaneus is a possible harvest site, with the iliac crest being superior to the other sites in terms of quality and quantity [77]. The literature concerning BMAC in GHOA is limited. Centeno et al. [53] showed encouraging results following BMAC injections for GHOA and rotator cuff pathology. Despite being considered a safe procedure, undesirable side effects can occur, particularly at the harvest site, such as chronic pain or nerve injury [77]. For this reason, Hernigou et al. [78] divided the iliac crest into sectors to direct trocars away from neurovascular structures. However, Centeno et al. [53] reported only five adverse events, none of which were serious, with only three cases of pain possibly related to the harvest procedure. Lastly, MSCs possess unique regeneration and anti-inflammatory properties, and they can be harvested from various sources, including bone marrow, adipose tissue, umbilical cord, and placental tissue [17, 24, 73, 77]. Adipose-derived MSCs are the most commonly collected because they are easily accessible [73, 77]. However, studies concerning their use in GHOA are scarce [17, 24, 73, 77]. Indeed, in this investigation, only one study by Fan et al. [53] evaluates the efficacy of autologous microfragmented adipose tissue treatment for shoulder or knee OA, and they found a significant improvement in multiple patient-reported outcome measures (PROMs) from two to 12 weeks and maintained weeks later. Despite the increased interest in these new biologics, no definitive conclusions can be drawn regarding the effect of either the BMAC or MSCs on GHOA [17, 24, 73, 77]. In the end, it is worth mentioning that, as for all intra-articular injections, side effects such as transient joint pain or systemic complications such as nausea, vomiting, and dizziness could occur [16, 77, 79, 80]. Furthermore, Eliasberg et al. [80] reported complications happening after injections of biologics, such as infections and suspected sterile inflammatory responses.

The present systematic review encountered substantial limitations given the inconsistent reporting of surgical interventions and complications across studies. Shoulder surgeries were conducted in five of the 13 studies [41, 51–62]. The highest revision rate was observed in the study by Kirschner et al. [55], where 16 patients ultimately underwent TSA, including ten during the study and six after the 12-month follow-up. In Merolla et al. [57], five patients in the HA group required surgery (four TSA, one arthroscopy). Metzger et al. [58] reported that 12 shoulders underwent repeat injections or arthroplasty, and Silverstein et al. [61] documented one case of TSA for persistent pain. Monti et al. [59] described a single surgical referral for unresolved symptoms. In the remaining studies, no revision procedures were reported. Adverse events were generally rare, mild, and transient, most commonly involving injection-site pain or arthralgia. No study reported serious treatment-related complications. A few studies noted isolated events such as cardiac symptoms or unrelated systemic complaints, but none were definitively attributed to the injection therapy. Surgical procedures were frequently documented as binary events without detailing indications, technical approaches, or postoperative rehabilitation protocols and outcomes. Similarly, complications were often aggregated into broad categories, obscuring critical differences in severity and management requirements. Superficial infections treated with oral antibiotics were conflated with deep joint infections that necessitated surgical debridement, whilst transient hemarthrosis cases were grouped with persistent synovitis that required intervention. Only a few included studies provided data on complication management timelines or escalation pathways. This reporting shortfall extended to surgical outcomes, where functional recovery metrics were rarely stratified by procedure type; arthroscopic debridement outcomes were indistinguishable from those of open capsular releases in pooled analyses. Consequently, the review could not evaluate whether specific injection-related complications predisposed patients to particular surgical interventions or vice versa. Another critical limitation of this systematic review stems from the substantial heterogeneity in pharmacological compounds and functional outcome measures across included studies. The analysed literature encompassed a diverse range of intra-articular agents, including HA and steroids, which possess distinct mechanisms of action, therapeutic indications, and durations of efficacy. This pharmacological variability directly influenced outcome reporting, as HA studies often emphasised mid-term pain relief and functional improvement (e.g. 6-month follow-ups). At the same time, CCs trials prioritised acute symptom reduction. Compounding this issue was the use of disparate functional assessment tools, such as the American Shoulder and Elbow Surgeons (ASES) score, Constant-Murley Shoulder Outcome Score (CMS), and Shoulder Pain and Disability Index (SPADI), which employ unique weighting systems for pain, mobility, and daily function. These methodological inconsistencies precluded meaningful meta-analysis of functional outcomes, as aggregation would have introduced significant measurement bias. Complications were often reported as aggregated categories without distinction between severity, management, or causal relationship, thereby precluding meaningful subcategorisation. The term’ ‘infiltrative management’ was deliberately preferred over ‘intra-articular injections’, as several of the included studies did not explicitly report the use of image-guided techniques to ensure accurate intra-articular delivery. Although this choice may appear overly cautious, it reflects the methodological heterogeneity of the available literature. Nevertheless, it should be emphasised that all authors referred to intra-articular administration, which is a prerequisite for the treatment of glenohumeral osteoarthritis. A further limitation of this review is the absence of a predefined protocol to resolve potential disagreements between reviewers, and no formal inter-author agreement was assessed. Although discrepancies were addressed through discussion, this methodological shortcoming may have introduced a source of subjectivity. These limitations highlight the need for standardised reporting frameworks in future studies, particularly about pharmacological protocols, outcome measurement tools, and adverse event classification. Addressing these gaps would enable more robust comparative analyses and enhance clinical translation of findings.

## Conclusion

Infiltrative management can provide symptomatic relief in adults with GHOA. Current evidence supports the potential role of different injectable therapies, with hyaluronic acid demonstrating consistent, though modest, benefits. In contrast, the evidence for orthobiologics remains limited, mainly because of heterogeneity in study design, outcome measures, and patient characteristics. High-quality comparative trials with long-term follow-up are required to establish optimal treatment strategies and to identify patient subgroups most likely to benefit from specific interventions.

## Supplementary Information

Supplementary material 1.

## Data Availability

The datasets generated during and/or analysed during the current study are available throughout the manuscript.
