# Supplementary material for: Intra-articular injections for shoulder arthritis in adults: a systematic review
Source: Eur J Med Res. 2025 Nov 7;30:1080. doi: 10.1186/s40001-025-03423-4 (PMC12593904; doi:10.1186/s40001-025-03423-4)
Supplement: Supplementary file 1 — Supplementary material 1. [file 40001_2025_3423_MOESM1_ESM.docx]

**Systematic literature search for:**

**Management of ailments of the shoulder cartilage in adults: A Systematic Review**

**Concept 1: Glenohumoral Cartilage leassons in adults**

**Keywords:**

Shoulder

Glenohumeral joint

Shoulder injuries

Cartilage

Cartilage lesion

Chondral defect

Osteochondral

Glenohumeral osteoarthritis

**Mesh:**

"Shoulder Joint"[Mesh]

"Shoulder Injuries"[Mesh]

"Cartilage, Articular"[Mesh]

"Cartilage Diseases"[Mesh]

### Concept 2: Intervention (non-arthroplasty, arthroscopic approches)

**Keywords:**

Arthroscopy/Arthroscopic

Injection

Hyaluronic acid (HA)

Hyaluronic acid infiltrations

HA infiltrations

HA injections

Intra-articular hyaluronic acid

Viscosupplementation

Non-animal stabilized hyaluronic acid (NASHA)

Corticosteroids (CCs)

Placebo

Platelet-rich plasma (PRP)

Mesenchymal stem cells (MSCs)

Conservative treatment

**Mesh:**

"Hyaluronic Acid"[Mesh]

“Platelet-Rich Plasma”[Mesh]

“Mesenchymal Stem Cells”[Mesh]

“Bone Marrow Cells”[Mesh]

**Exclusion Criteria: Arthroplasty or open surgery:**

**Keywords:**

Arthroplasty

Total shoulder arthroplasty

Reverse shoulder arthroplasty

TSA

Shoulder Replacement

Open surgery

**Mesh:**

“Rotator cuff”[Mesh]

“Frozen shoulder”[Mesh]

“Adhesive capsulitis”[Mesh]

“Instability”[Mesh]

“Arthroplasty, Replacement, Shoulder”[Mesh]

“Shoulder Prosthesis”[Mesh]

“Rotator Cuff Injuries”[Mesh]

“Shoulder Impingement Syndrome”[Mesh]

“Shoulder Dislocation”[Mesh]

“Bursitis”[Mesh]

**Concept 1:**

Shoulder Joint[Mesh] OR Shoulder Injuries[Mesh] OR Glenohumeral Joint[tiab] OR Cartilage, Articular[Mesh] OR Cartilage Diseases[Mesh] OR cartilage[tiab] OR chondral[tiab] OR osteochondral[tiab] OR cartilage lesion[tiab] OR chondral defect[tiab] OR glenohumeral osteoarthritis[tiab]

**AND**

**Concept 2:**

Hyaluronic Acid[Mesh] OR Platelet-Rich Plasma[Mesh] OR Mesenchymal Stem Cells[Mesh] OR Bone Marrow Cells[Mesh] OR injection*[tiab] OR hyaluronic acid[tiab] OR PRP[tiab] OR BMAC[tiab] OR bone marrow aspirate[tiab] OR stem cell[tiab] OR biologic therapy[tiab] OR nonoperative[tiab] OR non-surgical[tiab] OR conservative treatment[tiab]

**NOT**

Arthroplasty, Replacement, Shoulder[Mesh] OR Shoulder Prosthesis[Mesh] OR Rotator Cuff Injuries[Mesh] OR Shoulder Impingement Syndrome[Mesh] OR Shoulder Dislocation[Mesh] OR Bursitis[Mesh] OR Adhesive Capsulitis[Mesh] OR arthroplasty[tiab] OR total shoulder arthroplasty[tiab] OR reverse shoulder arthroplasty[tiab] OR TSA[tiab] OR shoulder replacement[tiab] OR open surgery[tiab] OR open[tiab] OR rotator cuff[tiab] OR frozen shoulder[tiab] OR adhesive capsulitis[tiab] OR instability[tiab]

**Summary of the Search**

((Shoulder Joint[Mesh] OR Shoulder Injuries[Mesh] OR Glenohumeral Joint[tiab] OR Cartilage, Articular[Mesh] OR Cartilage Diseases[Mesh] OR cartilage[tiab] OR chondral[tiab] OR osteochondral[tiab] OR cartilage lesion[tiab] OR chondral defect[tiab] OR glenohumeral osteoarthritis[tiab]) AND (Hyaluronic Acid[Mesh] OR Platelet-Rich Plasma[Mesh] OR Mesenchymal Stem Cells[Mesh] OR Bone Marrow Cells[Mesh] OR injection*[tiab] OR hyaluronic acid[tiab] OR PRP[tiab] OR BMAC[tiab] OR bone marrow aspirate[tiab] OR stem cell[tiab] OR biologic therapy[tiab] OR nonoperative[tiab] OR non-surgical[tiab] OR conservative treatment[tiab])) NOT (Arthroplasty, Replacement, Shoulder[Mesh] OR Shoulder Prosthesis[Mesh] OR Rotator Cuff Injuries[Mesh] OR Shoulder Impingement Syndrome[Mesh] OR Shoulder Dislocation[Mesh] OR Bursitis[Mesh] OR Adhesive Capsulitis[Mesh] OR arthroplasty[tiab] OR total shoulder arthroplasty[tiab] OR reverse shoulder arthroplasty[tiab] OR TSA[tiab] OR shoulder replacement[tiab] OR open surgery[tiab] OR open[tiab] OR rotator cuff[tiab] OR frozen shoulder[tiab] OR adhesive capsulitis[tiab] OR instability[tiab])

Filters applied: Clinical Trial, Randomized Controlled Trial.
